# Editorial: Dual disorders in addiction and mood disorders: comorbidity or specific diagnosis?

**DOI:** 10.3389/fpsyt.2024.1490922

**Published:** 2024-09-26

**Authors:** Warren B. Logge, Kunhua Lee, Cheng-Fang Yen

**Affiliations:** ^1^ Specialty of Addiction Medicine, Sydney Medical School, Faculty of Medicine and Health, University of Sydney, Sydney, NSW, Australia; ^2^ Edith Collins Centre for Translational Research in Alcohol, Drugs and Toxicology, Royal Prince Alfred Hospital, Sydney Local Health District, Sydney, NSW, Australia; ^3^ Department of Educational Psychology and Counseling, National Tsing Hua University, Hsinchu, Taiwan; ^4^ Department of Psychiatry, Kaohsiung Medical University, Kaohsiung, Taiwan

**Keywords:** dual disorders, substance use disorders, mood disorders, comorbidity, mental health interventions

Understanding dual disorders in addiction and mood disorders is crucial for providing comprehensive and effective care for individuals affected by these complex psychiatric conditions. Dual disorders, also known as co-occurring disorders, refer to the simultaneous presence of a substance use disorder and a mental health disorder within the same individual. The interplay between addiction and mood disorders, such as depression and bipolar disorder, is well-documented and represents a significant challenge in the field of psychiatry (1, 2). This Research Topic aims to explore the complexities of dual disorders, including their impact on diagnosis, treatment, and clinical outcomes.

Dual disorders in addiction and mood disorders encompass a wide range of psychiatric and medical conditions that can significantly influence the course of illness and treatment outcomes. Common co-occurring psychiatric disorders include anxiety disorders, personality disorders, and post-traumatic stress disorder (PTSD), among others (3). Individuals with addiction and mood disorders may also experience comorbid medical conditions such as liver disease, cardiovascular disease, and metabolic syndrome, which can further complicate their overall health and wellbeing (4). The presence of dual disorders has important implications for diagnosis and treatment. The overlapping symptoms and shared risk factors between addiction and mood disorders can pose challenges for accurate differential diagnosis, potentially leading to delayed or misdiagnoses. For example, symptoms of substance use can intersect with the mood symptoms of depression and bipolar disorder, complicating the identification of specific and dual disorders (5).

Several key themes emerge from this collection of articles, highlighting the intricate relationship between mood disorders and substance use disorders and the necessity for integrated treatment approaches. These articles emphasise the bidirectional influence between these conditions, where each can exacerbate the other, complicating diagnosis and treatment. The self-medication hypothesis is also explored, suggesting that individuals with mood disorders might use substances to alleviate their symptoms. Additionally, the role of psychosocial factors, such as social support, stress, and trauma, is examined to understand how they contribute to the development and persistence of dual disorders. Advances in genetic and biological research are presented, offering promising avenues for precision medicine and more personalised treatments.

The case report by Halim et al. focuses on a 21-year-old male with bipolar II disorder who developed a manic episode after using psilocybin mushrooms. This incident is significant as it may be the first documented case linking psilocybin to mania in bipolar II manifestation, highlighting the potential risks of psychedelic substances in this population. This case underscores the intricate relationship between substance use and mood disorders, and emphasizes the necessity for careful screening of bipolar risk when considering psychedelic treatments. Furthermore, it illustrates how substance use can exacerbate underlying mood disorders, complicating diagnosis and treatment. Overall, the report provides crucial clinical insights that contribute to understanding the management of dual disorders, particularly the need for further research to develop effective diagnostic and treatment strategies for patients with co-occurring substance use and mood disorders.

Wang et al.’s study examines the relationships between three types of sexual stigma, problematic Internet use and depression among a large cohort of young adults among sexual orientation diverse populations. A key finding of the study highlighted that individuals identifying as lesbian, gay, and bisexual with comorbid problematic Internet use and depression reported higher levels of internalized sexual stigma and sexual orientation microaggressions compared to individuals reporting only depression or only problematic internet use alone. This emphasises the significant impact of internalized stigma and microaggressions on mental health in among these young adults from sexual orientation diverse populations. It further highlights the importance of considering sexual stigma in the assessment and treatment of identifying individuals from these diverse populations with comorbid problematic internet use and depression.

Moulis et al. explored the prevalence and risk factors for depression among people who inject drugs in Vietnam. They identified several key risk factors for depression in this population, including being female, lacking permanent residency, frequent methamphetamine use, hospitalization, being single, and not having health insurance or being on methadone treatment. The study highlights the high prevalence of depression among people who inject drugs and underscores the need for targeted psychiatric interventions. The authors suggest that addressing social and structural vulnerabilities, such as housing instability and lack of social support, along with encouraging increased use of methadone maintenance therapies could be crucial in mitigating depression risk among people who inject drugs. These findings emphasize the importance of developing innovative prevention and care strategies tailored to the specific needs of this vulnerable population.

Lebiecka et al. investigated the impact of personality traits and depressive symptoms on behavioural control in patients with alcohol use disorders. Conducted with 53 inpatients at an alcohol rehabilitation centre, they examined the role self-control factors including impulsivity and cognitive response inhibition. Key findings indicated that personality traits such as conscientiousness and intellect, along with depression levels, significantly predicted impulsivity and processing speed. However, no significant link was found between impulsivity and cognitive response inhibition, suggesting they may represent distinct psychological constructs. This study highlights the importance of considering personality and depression in understanding and managing behavioural control in AUD patients, potentially guiding more tailored therapeutic approaches.

In conclusion, this Research Topic provides valuable insights into the complex interplay between addiction and mood disorders, highlighting the multifaceted nature of dual disorders. The studies presented underscore the importance of a holistic approach to diagnosis and treatment, considering not only the primary disorders but also the various factors that influence their development and progression. The studies in this Topic collectively emphasize the need for integrated, multidisciplinary approaches to treating dual disorders—through considering individual differences, social factors, and specific substance use patterns. They also highlight the importance of continued research to develop more effective diagnostic tools and treatment strategies for individuals with co-occurring substance use and mood disorders. By addressing these complex interactions, clinicians and researchers can work towards improving outcomes and quality of life for those affected by dual disorders.
